# Religious and traditional beliefs and practices as predictors of mental and physical health outcomes and the role of religious affiliation in health outcomes and risk taking

**DOI:** 10.1186/s12889-023-17030-7

**Published:** 2023-11-06

**Authors:** Ursula Wüthrich-Grossenbacher, Nicholas Midzi, Masceline Jenipher Mutsaka-Makuvaza, Abigail Mutsinze

**Affiliations:** 1 Center for African Studies, University of Basel, Rheinsprung 21, Basel, 4051 Switzerland; 2 National Institute of Health Research, Ministry of Health and Child Care, Harare, Zimbabwe; 3 Department of Microbiology and Parasitology, School of Medicine and Pharmacy, University of Rwanda, Kigali, Rwanda; 4 Africaid/Zvandiri, 11-12 Stoneridge Way North, Avondale, Harare, Zimbabwe

**Keywords:** HIV, Adolescents, Zimbabwe, Religion, Tradition, Viral load, Mental health, Risk taking

## Abstract

**Background:**

While many studies from sub-Saharan countries, including Zimbabwe, allude to the important role of religion and tradition for young people living with HIV (YPLHIV), most of these studies tend to be descriptive and lack advanced statistical analysis. This study aims to close this gap.

**Methods:**

Data for this study was collected between July and October 2021 from 804 YPLHIV (aged 14–24) in Zimbabwe. The questionnaire included questions regarding participants’ conceptions of HIV, their health seeking and risk-taking behaviour, current HIV viral load results, the prevalence of opportunistic infections, their mental health status, and demographic characteristics. The analysis was done using multilevel mixed-effects logistic regression.

**Results:**

We found that Traditional religious affiliation was linked to overall lower viral load (OR: 0.34; CI: 0.12–0.96; P: 0.042), Apostolic to more (OR: 1.52; CI: 1-2.3; P: 0.049) and Pentecostal to less (OR: 0.53; CI: 0.32–0.95; P: 0.033) treatment failure. Additionally, conceptions about HIV without spiritual or religious connotation, such as ‘seeing HIV as result of a weak body’ was associated with lower risk of treatment failure (OR: 0.6; CI: 0.4-1.0; P: 0.063), higher chances for undetectable viral load (OR: 1.4; CI: 1–2; P: 0.061), and overall lower viral load (OR: 0.7; CI: 0.5-1; P: 0.067). Moralizing concepts of HIV, like ‘seeing HIV as a result of sin in the family’, was linked to higher risk of opportunistic infections (OR:1.8; CI:1.1-3; P: 0.018), and higher risk of treatment failure (OR: 1.7; CI: 0.7–1.1; P: 0.066). Religious objections toward certain forms of therapy, like toward cervical cancer screening, was linked to higher risk of mental problems (OR: 2.2; CI: 1.35–3.68; P: 0.002) and higher risk of opportunistic infections (OR:1.6; CI:1.1–2.1; P: 0.008). Religious affiliations significantly influenced conceptions of HIV, health seeking behaviour, and risk taking.

**Conclusion:**

To our knowledge, this study is the first to provide evidence about the statistically significant associations between religious and traditional beliefs and practices and current health outcomes and health risk factors of YPLHIV in Zimbabwe. It is also the first to identify empirically the role of religious affiliations as predictors of current viral load results. This new knowledge can inform contextualized approaches to support YPLHIV in Zimbabwe.

## Introduction

### Background

According to UNICEF, Zimbabwe has an adult Human Immunodeficiency Virus (HIV) prevalence rate of 11.58%, corresponding to about 1.3 million Zimbabweans living with HIV. Of those, 77,300 are adolescents aged 10–19. Females aged 15–29 years have the highest HIV rate [1]. Alarmingly, the Zimbabwe *2015 Demographic Health Survey* recorded an increase in risk behaviors after 2010 including having sex with non-regular partners, having multiple sexual partnerships, and encounters with sex workers [2]. Thus, to reach the *Sustainable Development Goal 3* [3] target of ending the epidemic of Acquired Immune Deficiency Syndrome (AIDS) by 2030, new prevention and care approaches are needed to combat current trends and better support young people living with HIV (YPLHIV) in Zimbabwe.

Zimbabwe is a highly religious country. 84% of the population aged 15 years and above are Christians. The largest proportion of Christians belong to the Apostolic Sect. (34%), followed by Pentecostals (20%), and Protestants (16%). Muslims, Jews, Buddhists, Hindus, and New Religious Movements are minority groups in Zimbabwe [4]. Furthermore, according to Chitando, every African is born into African Traditional Religions and that influences the way Zimbabweans practice religion [5]. Besides, since around 2009, Zimbabwe has witnessed a surge of Christian preachers who call themselves prophets or prophetesses. They claim to be mediators between God and ordinary people, and profess to work miracles, including healings [6]. Many Zimbabweans freely combine or move between traditional religion and different Christian churches, including prophet led churches. Health and illness are not only understood as physical phenomena but seen and understood in that traditional and religious context. Thus, illness may have physical, mental, social, spiritual, and supernatural causes. This is true for all African ethnic groups in Zimbabwe (Shona 82%, Ndebele 14%, others). Many studies describe the important influence of traditional and religious practices and beliefs on health seeking behaviour, suggesting that religion and tradition could play an important role in providing additional support structures that facilitate treatment adherence. However, most of these studies are qualitative in nature, or based on descriptive statistics. Given the significant gap in our empirical knowledge, it is unsurprising that UNICEF called for future studies to apply multi-level logistic regression to test and reveal the strengths of different causes [7].

### Aims

The aim of this study is to respond to the lack of evidence from inferential statistical analysis by using multilevel mixed-effects logistic regression to explore whether it is possible to identify traditional and religious practices and beliefs that significantly influence the current viral load, the mental health status, the prevalence of opportunistic infections, and the level of risk behaviour of YPLHIV in Zimbabwe. The study also explores the role of religious affiliation in relation to health risk factors and health outcomes.

### Summary of existing literature

The influence of religion and tradition on health and health seeking behaviour is widely debated globally as well as in Zimbabwe: In 2021, Mapingure et al. used the 2015–2016 *Zimbabwe Demographic and Health Survey* to investigate the understanding of HIV and associated risk factors among religious groups in Zimbabwe [8]. They found that, compared to other religious groups, members of the Apostolic religion lacked adequate knowledge of HIV and associated risk factors. These findings are in line with UNICEF’s extended analysis of the Zimbabwe *Multiple Indicator Cluster Survey* released in 2014 [7]. In the latter, UNICEF analyzed the influence of different religions on health, educational, and social outcomes. They found that members of the Apostolic, Traditional, and No-religion (those without religious affiliation) all belonged to the poorer section of the population and had higher health risks. Several other studies complemented these findings by examining the link between religious (mainly Apostolic) affiliation and poor(er) health seeking behaviour [6, 7]. A study conducted by Nhamo and Murire, for example, stressed the importance of beliefs, internalized norms, stigma, and religion [9]. This was exemplified by Mutambare et al. who found that, in Gweru (an ordinary town in the center of Zimbabwe), people living with HIV defaulted on medication use because they believed in faith healing, alternative medicines, and perceived spirituality as the main cause of HIV and AIDS [10]. Other studies are limited to describing the role of religious stigma [11], religious norms associated with sexuality and gender [9, 10, 12], and the belief in witchcraft as the spiritual source of illness and health [13]. Finally, Shoko points to the significance of traditional beliefs and practices by explaining that traditional medicine is important to Shona people because it addresses aspects which bio-medical practice fails to [14]. According to him, Shona people conceive and practice healing holistically by embracing physical, spiritual, psycho-emotional, social, and ecological dimensions. Accordingly, we can trace three independent, parallel health systems in Zimbabwe: biomedical, traditional, and religious [15]. Our own scoping review identified at least three areas of conflict between these disparate health practices relating to the bio-psycho-socio-spiritual understanding of health and illness, the notion of patriarchy, and the perception of sexuality and procreation [16]. Given these findings, local scholars have called for strategies that facilitate the integration of the indigenous health system to improve adolescent sexual health outcomes [14, 15]. We also support this but argue to expand this further to also include religious stakeholders. In this study, we therefore explore the role of religious and traditional beliefs and practices to gain a better understanding of the impact of religion/spirituality and tradition on the health and wellbeing of YPLHIV in Zimbabwe.

### Contribution to the field

This is the first study in Zimbabwe that sought to identify belief-based concepts of HIV and HIV treatment with statistically significant links to current viral load results, mental health, and opportunistic infections. It is also the first study that examines the relationship between religious affiliations and health outcomes and health risk factors. These insights provide important new evidence to inform religiously sensitive and contextually relevant additional interventions and support structures to boost treatment adherence of YPLHIV.

## Methods

All participants were beneficiaries of Zvandiri’s peer support program. Zvandiri connects children and young people living with HIV with trained, mentored peers to improve their mental and physical wellbeing [17]. The study population included 804 eligible antiretroviral therapy (ART) program clients of both sexes from Mt.Darwin, Seke, Mazowe, Mberengwa, Harare, Bulawayo, and Beitbridge district located in urban, peri-urban, and rural areas in Zimbabwe. The study sites were purposefully chosen according to the obtainability of current valid viral load results, and to ensure a balance of locations, languages, and cultures (Shona, Ndebele, English), dominant religious environments (Apostolic sects, main churches, traditional), and health facility structures (difference in user fees). The inclusion criteria were: YPLHIV in an ART program; aged between 14 and 24 years; willing and able to provide informed consent, or willing and able to provide assent, if aged below 18 and consented first by caregiver; and availability of a viral load result not older than 12 months. Zvandiri personnel identified eligible candidates who were then contacted by the data collectors. About 10% of identified eligible candidates were not reachable by phone. This was mainly due to the hardship situation during Covid-19 lockdown. Electricity was often interrupted, phone connectivity challenged, and often people did not have money to buy cards for their phones. This situation was the same for all beneficiaries. All people who met the inclusion criteria, and were reachable by phone, were included in the study. Thus, there was no selection bias. Recruitment was stopped after we reached the desired number of participants.

The questionnaire contained the following baseline data: Questions regarding the understanding or conception of HIV including beliefs about God, beliefs about spirits, and conceptions about the origin and causes of HIV; questions regarding participants’ health seeking behaviour, including attitudes towards certain therapy forms, the types of healers consulted or planning to consult, and the traditional medicines and religious/traditional practices participants rely on; questions regarding risk behaviour, including potentially harmful substance use and sexual risk behaviour, and the attitude towards and the experience of violence; religious affiliations (multiple answers possible); current health outcomes measured in viral load result (participants took the information from their health record), mental health status (using the *Shona Symptom Questionnaire*) [18], and the prevalence of current/past opportunistic infections (participants chose from a list with 15 different infections including TB, Toxoplasmosis, Candidiasis, Skin problems, etc. and also had the option ‘other’, ‘don’t know’, or ‘none’); and demographic characteristics (age, location, education, civil status, and income). Red flags for answers indicating the need for psychological or legal intervention were strategically built into the questionnaire and participants who were flagged were referred to the Zvandiri counselling team [19]. The English questionnaire was professionally translated into the two major local languages, Shona and Ndebele. Zvandiri recruited the data collection team from their peer community adolescent treatment supporters [17]. After data collectors completed intensive training, a successful pilot study was conducted in May 2021. The quantitative data collection was conducted with the Open Data Kit (ODK) questionnaire between July and October 2021. Due to the COVID-19 pandemic, most of the questionnaires had to be administered by phone, some (96) were administered face to face, and about 40 by ODK self-administration via public link. According to requirements stipulated by the Medical Research Council of Zimbabwe, all participants were compensated with five US Dollars and reimbursed for their airtime required to participate in the study.

The aim of this study was to identify possible and relevant associations between traditional and religious practices and beliefs and viral load, mental health status, and the prevalence of opportunistic infections. Additionally, we explored relations between religious affiliation, health outcomes, and health risk factors.

The data was analyzed using the statistical software STATA version 17.0 (Stata Corp, College Station, TX, USA). Frequency distributions were calculated using tabulation. The statistical analysis was conducted using multilevel mixed-effects ordered logistic regression and multilevel mixed-effects logistic regression for binary outcome variables. The cut-off for the *P*-value was set at 0.1 and 0.05 for significant relations. The regression controlled for mental health, age, gender, education, and location (= health facility).

### Sociodemographic results

The study included 804 participants who were all beneficiaries of Zvandiri’s peer support program [17]. The viral load results of two participants were still pending when doing the analysis and they had to be excluded, resulting in a total of 802 participants who were included in the analysis. The sociodemographic of the participants is listed below (Table 1).

**Table 1:** Participants’ sociodemographic

Demographic Variable	Category 1	Category 2	Category 3
Age	14–15 years: 15%	16–17 years: 45%	18–24 years: 40%
Gender	Females: 61.3%	Males: 38.4%	Other: 0.3%
Location	Urban: 49%	Peri-urban: 13%	Rural: 38%
Monthly income for (> 18 years)	< 25 USD: 86%	Between 25–150 USD 11%	> 150 USD: 3%
Education completed	< Grade 7: 7.1% Grade 7: 20%	Secondary: 65.4%	A-Level: 5% Tertiary: 2.5%
Relationship status	Single: 65.4%	Partner/Married: 33.4%	Divorced/Widowed: 1.2%

This table is slightly adapted from one of our previous publications [20]

### Religious affiliations

In this paper, the term “religion” refers to organised and/or shared faith practices or beliefs and the term “spirituality” refers to the way people relate to the transcendent, including traditional practices. In the questionnaire, participants were asked, “what is your religious affiliation?” and participants were allowed to choose multiple religious affiliations. Of these, Apostolic affiliation was the most frequently reported. Females had a remarkably higher percentage of Anglican, Methodist, Muslim and no religious affiliation, and males reported remarkably higher rates of Traditional and Apostolic religious affiliation (Table 2). Of the two participants who chose ‘other’ gender, one chose Methodist and one Pentecostal religious affiliation.

**Table 2:** Religious affiliation by gender

Personal Religion	Male	Female	Total
Apostolic	25.65% (79)	23.58% (116)	24.31% (195)
Pentecostal	20.45% (63)	18.5% (91)	19.33% (155)
Other	19.81% (61)	18.7% (92)	19.8% (153)
none	8.77% (27)	10.98% (54)	10.1% (81)
Methodist	5.84% (18)	9.55% (47)	8.23% (66)
Catholic	7.79% (24)	6.1% (30)	6.73% (54)
Traditional	5.52% (17)	2.44% (12)	3.62% (29)
more than one religion	2.92% (9)	2.85% (14)	2.87% (23)
Anglican	1.3% (4)	3.46% (17)	2.62% (21)
Baptist	1.62% (5)	1.63% (8)	1.62% (13)
Muslim	0.32% (1)	1.83% (9)	1.25% (10)
Prefer not to answer		0.4% (2)	0.25% (2)

Number of participants in brackets

### Descriptive results

#### The understanding and conceptions of HIV

Faith in God was central in the life of most participants. More than three quarters of participants reported that they had no doubt about the existence of God and one fifth that their relationship with God was their priority in life. Beliefs regarding spirits/spells were less prominent and more diverse. One third of participants said that they did not believe in the existence of spirits, and that spirits/spells had no power. However, only 7% said that ill health is never caused by spirits. One quarter said they believed that spirits/spells have power and 6% that ill health was always caused by spirits/spells. Some claimed that spirits/spells influenced them in the past (7%) or are influencing them now (3%). The beliefs about the origin of HIV were similarly diverse. Participants chose a combination of traditional/cultural, and spiritual/religious explanations. Participants were further asked why some people get infected with HIV and others do not. Most common were moralizing concepts of HIV (Table 3).

**Table 3:** Beliefs about Origin of HIV and reasons why some people get infected and others don’t

**Beliefs about Origin of HIV**	**Number**	**%**
Don’t know	203	25
HIV originated because of mixing of blood (different races, different age groups)	175	22
HIV was given by God as punishment for sexual misconduct	159	20
HIV was brought by the West to weaken Africans	119	15
Other reasons	111	14
HIV came from homosexuals	100	12
HIV was caused by a foreign chemical substance	84	10
HIV always originates with women	17	2
**Belief why some get infected by HIV and others don’t**		
HIV is the result of immoral sexual behaviour in the family or self	338	42
Weak body	200	25
Don’t know	183	23
Personal sin	99	12
Bad luck	86	11
Result of sin in the family	75	9
Result of witchcraft / spell	36	4.5
Grieving of ancestors	31	3.9

Thus, for most participants, the belief in God and the perception of a transcendent influence on health are embedded in the experience of living with HIV and inform their health seeking behaviour.

#### Health seeking behaviour

Because HIV and HIV related illnesses are not merely seen as physical problems, but as having a spiritual or transcendent dimension, half of YPLHIV in our study wished to choose their health practitioner according to the cause of their illness and many freely moved between or within the three health systems (religious, traditional, biomedical). 40% of participants consulted religious healers. Of these, prophets (18%) were consulted most frequently. Two thirds of participants used religious rituals, of which prayer was the most common. 28% used prayer for healing and 14% used prayer for forgiveness. The following reasons were provided for consulting traditional healers: because they help holistically (physically and spiritually); they know the culture; they have access to ancestors; and they are affordable. Besides health concerns, traditional healers are consulted for various other issues including the preservation of morality, good luck charms, and dealing with relationship issues. Also, the use of herbal supplements was common, with two thirds of participants taking at least one supplement, and the highest reported number being seventeen different supplements. According to participants, the affordability of traditional medicine, especially relative to western medicine, is the primary reason for using it. Other important reasons included their accessibility, their natural/African origin, and because they are viewed as effective.

The parallel use of the biomedical, religious, and traditional health systems can lead to conflicting and compromising treatment approaches. This has also been found here: 10.5% of participants reported having at least once stopped taking ARVs for religious reasons. The following reasons were provided for having stopped ARVs: belief God/spirit would heal (42%); not wanting religious people to know that they are on ARVs (10%); belief that ARVs are not part of their culture/belief (9%); told by religious people to stop (6.3%); and belief that God was punishing them (4%). Besides these, the participants were also confronted with other religious reasons that oppose certain forms of treatment. 32% knew religious reasons that spoke against traditional medicine and 29% against western medicine. 26% had heard of or knew religious reasons that spoke against cervical cancer screening. Interestingly, the latter also had higher screening rates. The divergence between religious doctrine that speaks against (western) cervical screening and actual health seeking behavior highlight the complexity of the religion-health context within which participants operate. Not only do YPLHIV need to navigate three – at times - contrasting health systems, but additionally they are confronted with opposing views and approaches. Unsurprisingly, one third of participants wished that all healers (medical, traditional, religious) worked together. The importance of this wish is further underlined by our findings regarding risk behaviours, that highlight additional points of conflict, especially in regard to sexual practice.

#### Risk behaviours

UNICEF recently pointed out that child marriage is very common in Zimbabwe and that one in three women marry before the age of 18 [21]. Among our participants, one third of participants started sexual activity before the age of 17, 17 (2.1%) were married before the age of 18, and 6 participants were sexually active before the age of 12. 41 (5.1%) participants (34 females, 7 males) younger than 18 had at least one child. 27% of all females reported at least one pregnancy and 6% of sexually active females had at least one abortion. Condom use was not popular, as less than half of those who reported being sexually active used a condom all the time. The higher the number of lifetime sexual partners, the higher the percentage of those who never use a condom. Condom use was slightly higher for males than for females. Furthermore, about one quarter of those who are sexually active had a minimum of five lifetime sexual partners and 11.4% had at least once sexual encounter with a person of the same sex.

In regard to the perception and experience of violence, 34% of participants experienced some form of non-sexual violence. Regarding non-sexual violence at home, nearly three quarters of respondents condoned the beating of children under certain circumstances. Much less accepted was beating one’s wife. In this instance, 73% of participants did not accept wife beating. Some however, saw some legitimate reasons for beating: arguing with husband (12%), going out without telling the husband (10%), neglecting their children (6%), or refusing sex (6%).

7% of participants reported that they had experienced sexual violence, while some preferred not to answer. Data collectors assumed that the majority of those responding with “don’t know” or “prefer not to answer” felt too uncomfortable to describe their experiences. However, due to the uncertainty associated with making this assumption, the “don’t know” and “prefer not to answer” answers were coded as “no” in the binary variable ‘experienced sexual violence’ that was used for the regression analysis. In our study, there was only a small difference between genders.

Relative to other risk behaviours, participants reported the use of potentially harmful substances as follows: 7.5% of participants smoke, 15.6% of participants reported the consumption of alcohol, and 4.9% reported the use of drugs. Again, the data collectors felt that these relatively low numbers may be due to a perceived lack of privacy on the side of the respondents, who at times had to use their parents’ phone and were confined to their homes because of Covid-19.

#### Health outcomes

The viral load result was described with three different variables, using the *European Aids Clinical Society*’s guidelines [22]: Viral load with three different values corresponding to undetectable viral load (≤ 50 copies/ml), incomplete suppression (> 50 copies/ml ≤ 200 copies/ml), and treatment failure (> 200 copies/ml); TND (binary for undetectable viral loads ≤ 50 copies/ml); and Failure (binary) for viral load results indicating treatment failure (> 200 copies/ml). Nearly three quarters of all participants had an undetectable viral load, suggesting good treatment adherence. However, 16% of participants had a viral load that suggests poor treatment adherence and treatment failure (Table 4). Out of the 804 participants only two had pending viral load results, all other viral load results were dated within 5 months of data collection.

42.8% of participants had at least one opportunistic infection. 9.2% had an average score of more than 8 in the *Shona Symptom Questionnaire*, indicating a risk for mental health problems (Table 4).

**Table 4:** Viral load results, prevalence of opportunistic infections, mental health risk

Variable Name	Result	Definition
Viral load (Three values 1–3)	74% 10% 16%	≤ 50 copies/ml > 50 copies/ml ≤ 200 copies/ml > 200 copies/ml
TND (binary)	74%	≤ 50 copies/ml
Failure (binary)	16%	> 200 copies/ml
Prevalence of opportunistic infections (binary)	42.8%	≥ 1 opportunistic infection
Mental Health risk (binary)	9.2%	> 8 in Shona Symptom Questionnaire (mean of 14 items)

### Regression results

#### The understanding and conceptions of HIV

Belief-based conceptions about the origin or reason for HIV had relevant relations to health outcomes and as such acted as facilitators or barriers to good health. Concepts without spiritual or religious connotation were linked to better health outcomes, while moralizing concepts of HIV were linked to a higher risk of opportunistic infections. This was also the case for the belief that HIV was brought by the West to weaken Africans. Seeing HIV as a result of witchcraft/spells was additionally linked to a higher risk of mental health problems (Table 5).

**Table 5:** Concepts of HIV with relevant associations with health outcomes

Concept of HIV (binary)	Good Health Facilitator	Good Health Barrier
Not believing in the existence of spirits	**Less opportunistic infections **(OR:0.7; CI:0.5-1; P: 0.037).	
Only physical origin “weak body”	Lower risk for treatment failure (OR: 0.6; CI: 0.4-1.0; P: 0.063)	
	Higher chance for TND (OR: 1.4; CI: 1–2; P: 0.061)	
	Lower viral load (OR: 0.7; CI: 0.5-1; P: 0.067)	
Brought by the West to weaken Africans		More Opportunistic Infections (OR: 1.5; CI: 1-2.2; P: 0.061)
Result of immoral sexual behaviour in the family or self		**More Opportunistic Infections **(OR:1.6; CI:1.2-2; P: 0.004)
Result of sin (family)		**More Opportunistic Infections **(OR:1.8; CI:1.1-3; P: 0.018)
		Higher risk of treatment failure (OR: 1.7; CI: 0.7–1.1; P: 0.066)
Result of witchcraft / spells		**More Opportunistic Infections **(OR:2.3; CI:1.1–4.6; P: 0.026)
		**Higher risk of mental problems **(OR: 2.6; CI: 1-6.3; P: 0.039)

The relationship between each concept of HIV (independent variable) with each of the health parameters (dependent variable) was individually calculated with multilevel mixed-effects logistic regression controlling for gender, age, education, and location. Each row shows individual models. Only significant (*P* value less than 0.05) and moderate (*P* value between 0.05 and 0.09) relations between independent variable and dependent variable are reported. Significant relations are bold. Each row of the table represents one regression model with the outcome as listed on the right

#### Health seeking behaviour

Participants who experienced religious objections toward certain forms of therapy had a higher risk of mental health problems and in most cases also a higher prevalence of opportunistic infections. The consultation of religious and traditional healers was not related to health outcomes, except for those who consulted herbalists. None of the herbs had a direct significant link to health outcomes. However, those who consumed many different herbs had a higher prevalence of opportunistic infections (Table 6).

**Table 6:** Health seeking behaviour with relevant associations with health outcomes

Beliefs about right treatment (binary) Ever heard religious reasons …	Good Health Facilitator	Good Health Barrier
against Cervical Cancer Screening		**Higher risk of mental problems **(OR: 2.2; CI: 1.35–3.68; P: 0.002)
		**More Opportunistic Infections **(OR:1.6; CI:1.1–2.1; P: 0.008)
against western Medicine		**Higher risk of mental problems** (OR: 2.4; CI: 1.28–3.98; P: < 0.001)
		**More Opportunistic Infections **(OR:1.4; CI:1–2; P: 0.031)
against traditional Medicine		**Higher risk of mental problems **(OR: 2.2; CI: 1.35–3.65; P: < 0.002)
Consultation of Healers: Herbalists		**Higher risk of mental problems **(OR: 4.2; CI: 1.7–10.2; P: 0.002)
Using of traditional medicine		The higher the number of herbal supplements the **more opportunistic infections** (OR:1.1; CI:1–2; P: 0.031)

The relationship between each health seeking behaviour variable (independent variable) with each of the Health Outcome variable (dependent variable) was individually calculated with multilevel mixed-effects logistic regression controlling for gender, age, education, and location. Only significant (*P* value less than 0.05) and moderate (*P* value between 0.05 and 0.09) relations between independent variable and dependent variable are reported. Significant relations are bold. Each row of the table represents one regression model with the outcome as listed on the right

#### Risk behaviours

Risk behaviours did not significantly relate to viral load, but some significantly increased the prevalence of opportunistic infections (Table 7). In terms of sexual risk behaviours, the higher the age at which respondents first initiated sexual activity, the lower the risk of sexual violence, (OR: 0.8; CI: 0.7-1.0; P: 0.098) and the lower the number of sexual partners (OR: 0.8; CI: 0.7–0.9; P: 0.0).

Besides a higher prevalence of opportunistic infections, the experience of violence was additionally related to a higher risk of mental health problems (Table 7). Accepting certain reasons for beating was linked to a higher prevalence of opportunistic infections, while the belief that beating is never justified was significantly linked to a lower risk of opportunistic infections (OR 0.7; CI 0.5–0.9; P 0.019).

**Table 7:** Risk behaviours with relevant associations to health outcomes

Risk Behaviour (binary)	Good Health Facilitator	Good Health Barrier
Substance use Smoking		**More Opportunistic Infections** (OR:2.6; CI1.5-4.8:1.5–4.8; P: 0.001)
Alcohol		Less TNDs (OR:0.7; IC: 0.5-1.0; P: 0.090)
		**More Opportunistic Infections** (OR:1.6; CI:1-2.4; P: 0.017)
Drugs		**More Opportunistic Infections** (OR: 2; CI: 1-4.2; P: 0.05)
Sexual risks Minor Sex		**More Opportunistic Infections** (OR:1.5; CI:1–2; P: 0.021
No condom		**More Opportunistic Infections** (OR:1.2; CI:0.7-1; P: 0.012)
Many sex partners	**Lower risk of mental problems** (OR: 0.85; CI: 0.7–0.99; P 0.041)	
		More Opportunistic Infections (OR:1; CI: 1-1.2; P 0.054).
Teenage Parenting		Higher risk of mental problems (OR: 2.5; CI: 0.9–6.5; P: 0.068)
Experience of non-sexual violence		**Higher risk of mental problems** (OR: 2.4; CI: 1.5–3.9; P: 0.001)
		**More Opportunistic Infections** (OR:1.8; CI:1.-2.5; P: < 0.001
Experience of sexual violence		**Higher risk of mental problems** (OR: 2.7; CI: 1.3–5.4; P: 0.005)
		**More Opportunistic Infections** (OR: 2.5; CI: 1.3–4.6; P: 0.004)

The relationship between each risk behaviour variable (independent variable) with each of the Health Outcome variable (dependent variable) was individually calculated with multilevel mixed-effects logistic regression controlling for gender, age, education, and location. Only significant (*P* value less than 0.05) and moderate (*P* value between 0.05 and 0.09) relations between independent variable and dependent variable are reported. Significant relations are bold. Each row of the table represents one regression model with the outcome as listed on the right

So far, the regression results identified that belief-based conceptions about HIV, religious objections towards certain forms of therapy, the reliance on traditional practice, and individual risk behaviours were significantly related to physical and mental health and thus acted as barriers or facilitators to good health. Next, we explored the relationship between religious affiliations and health outcomes. Does religious affiliation significantly influence health outcomes?

#### The relation between religious affiliation and factors acting as facilitators and barriers of good health

In our study, we found that people do not disclose their HIV status to those outside their close family and friendship circles. Nearly one fifth of the participants explicitly said they did not want church leaders and church members to know. Nevertheless, certain religious affiliations significantly influenced specific conceptions of HIV, health seeking behaviour, and risk behaviours that have shown to act as facilitators or barriers to good health. There are some shared associations with health barriers, especially by no religious affiliation and Traditional religious affiliation. Both are significantly linked to the use of potentially harmful substances, the condoning of wife beating, and sexual activity below the age of 17 years. Apostolic and Pentecostal religious affiliations are the only religious affiliations that significantly related to both, a good health facilitator and a good health barrier. Interestingly certain factors that act as barriers exclusively related to only one specific religious affiliation. For instance, having stopped ARVs for religious reasons only related to Traditional religion, and religious reasons against cervical cancer screening only related to no religious affiliation (Table 8).

We conducted individual multilevel mixed-effect logistic regressions (controlling for gender, age, education, and location) with a Good Health Facilitator or a Good Health Barrier as dependent variable and a religious affiliation as independent variable.

**Table 8:** Religious affiliations with significant associations with facilitators and barriers to good health

Religious affiliation	Good Health Facilitator	Good Health Barrier
Traditional		**Prevalence of having stopped ARVs for religious reasons **(OR:3.9; CI:1.7–9.2; P: 0.001)
		**Smoking **(OR:5.3; CI:2.4–11.7; P:0.0)
		**Alcohol consumption **(OR:2.9; CI: 1.4–5.9; P: 0.003)
		**Drug use **(OR:3; CI: 1-8.4; P: 0.04)
		**Condoning of wife beating **(because of burning food: OR 2.5; CI 1.0-6.1; P 0.05)
		**Sexual activity below 17 years **(OR: 2.1; CI: 1.0-4.2; P: 0.031)
Apostolic	**Reduced non-sexual violence experience** (OR 0.7; CI: 0.5–0.95; P: 0.026)	
		**Belief that HIV was given by God as punishment for sexual misconduct (OR: 1.5; CI: 1–2.2; P: 0.047)**
		**Condoning of wife beating **(for going out without telling: OR 1.6; CI 1.0-2.1; P 0.06)
Pentecostal	**Less smoking** (OR: 0.32; CI: 0.12–0.86; P: 0.024)	
		**Belief that HIV is the result of immoral sexual behaviour in the family or self (OR: 1.6; CI: 1.1–2.3; P: 0.008)**
Anglican		**Belief that HIV is the result of sin in the family **(OR: 5.9; CI: 2.5–13.9; P: < 0.001)
		**Experience of prostitution **(OR: 9.0; CI: 1.5–53.8; P: 0.016)
		**Increased number of lifetime sexual partners** (OR: 2.7; CI: 1.0-7.2; P 0.042)
Muslim		**Sexual activity below 17 years** (OR: 4.3; CI: 1.2–15.8; P: 0.026)
None		**Belief that HIV was brought by the West to weaken Africans **OR:( 2.2; CI: 0.7–1.2; P: 0.002)
		**Religious objections against cervical cancer screening** (OR: 1.7; CI: 1.0-2.9; P: 0.035)
		**Smoking **(OR:4.8; CI: 2.4–9.5; P: 0.0)
		**Alcohol consumption **(OR:3.7; CI 2.9–6.3; P: 0.0)
		**Condoning of wife beating **(always allowed: OR 4.4; CI 1.3–14.8; P 0.018)
		**Sexual activity below 17 years **(OR: 2.0; CI: 1.2-3-3; P: 0.006)
		**Increased number of lifetime sexual partners **(OR: 2.7; CI: 1.6–4.6; P < 0.001)

The relationship between each religious affiliation variable (independent variable) with each of the Good Health Barrier and Good Health Facilitator variable (dependent variable) was individually calculated with multilevel mixed-effects logistic regression controlling for gender, age, education, and location. Only significant (*P* value less than 0.05) relations between independent variable and dependent variable are reported. Each row of the table represents one regression model with the outcome as listed on the right

While these findings show the indirect relationship between religious affiliation and health outcomes, we also identified direct significant relations between religious affiliations and health outcomes.

#### Religious affiliation and health outcomes

Traditional, Pentecostal, and Apostolic religious affiliations had direct significant links with current viral load results and, in the case of Traditional religion, with the prevalence of opportunistic infections (Table 9). This means that religious affiliations may influence health outcomes in positive and negative ways. None of the religious affiliations were significantly linked to mental health. We conducted individual multilevel mixed-effect logistic regressions (controlling for gender, age, education, and location) with a Health Outcome variable as dependent variable and a religious affiliation as independent variable.

**Table 9:** Religious affiliations with significant links to viral load and opportunistic infections

Religious affiliation (binary)	Good Health Facilitator	Good Health Barrier
Traditional	**Lower viral load **(OR: 0.34; CI: 0.12–0.96; P: 0.042)	
	More TNDs (OR: 2.04; CI: 0.9–6.08; P: 0.082)	
		**More Opportunistic Infections **(OR: 2.2; CI: 1.1–4.4; P: 0.023)
Apostolic		**More treatment failure **(OR: 1.52; CI: 1-2.3; P: 0.049)
Pentecostal	**Less treatment failure **(OR: 0.53; CI: 0.32–0.95; P: 0.033)	

The relationship between each religious affiliation variable (independent variable) with each of the Health Outcome variable (dependent variable) was individually calculated with multilevel mixed-effects logistic regression controlling for gender, age, education, and location. Only significant (*P* value less than 0.05) and moderate (*P* value between 0.05 and 0.09) relations between independent variable and dependent variable are reported. Significant relations are in bold. Each row of the table represents one regression model with the outcome as listed on the right

In summary, our findings support the idea that YPLHIV in Zimbabwe make use of parallel, and often incongruent biomedical, traditional, and religious health systems. We also identified the statistically significant influence of religious and traditional beliefs and practices on mental and physical health and showed how different religious affiliations act as predictors of health outcomes.

## Discussion

To our knowledge, this was the first study in Zimbabwe that identified statistically significant associations of certain religious and traditional beliefs and practices with current health outcomes and health risk factors. It is also the first study to identify religious affiliations as predictors of physical and mental health outcomes, as well as risk behaviours, especially the predictive role of Traditional and Pentecostal religious affiliation on lower viral load and better treatment adherence (via less treatment failure), and the predictive role of Apostolic religious affiliation on higher risk of treatment failure. This demonstrates the pivotal role of faith communities and highlights the potential for collaboration between health professionals and religious and traditional leaders.

To elaborate on this, we would like to discuss and highlight three different issues:

First, for most participants, the belief in God and the perception of a transcendent influence on health are embedded in their experience of living with HIV and therefore inform their health seeking behaviour. The important role that religious and traditional beliefs and practices play in the lives of our study participants is observable in the significant rates of religious affiliation and (religious) health seeking behaviour (90% with at least one religious affiliation, 40% that consult religious healers, and 60% that use herbal supplements). Our findings indicate that the parallel use of the three different health systems results in conflicting and compromising approaches to care, and significantly also impacts on treatment outcomes. To work against mistrust and misconceptions, and to avoid belief-based objections from acting as barriers to accessing care, there is need for in- depth discussion within and between the different health sectors to find new, religiously sensitive, and contextually relevant solutions to address these issues.

Second, participants’ risk behaviours may not mirror official religious doctrine, but they are nevertheless linked with religious affiliations. The fact that different religious affiliations were associated with different factors that act as facilitators or barriers to good health, alludes to the importance of religious and traditional teaching and practice. However, the findings suggest that practice may be more important than doctrine. This was especially seen in the context of sexuality. While most religious and traditional teachings advocate sexual abstinence outside of marriage and condemn sexual risk behaviours, the study results reveal that a fair number of participants were sexually active before the age of 17, had more than five lifetime sexual partners, and presently engaged in sexual risk behaviours (no condom use, same sex relationships). This is of relevance for all religious and traditional leaders. It is time to acknowledge the discrepancy between preached doctrine and lived practice. The established links between religious affiliations and risk behaviours, including the condoning and experience of violence, require religious and traditional leaders to acknowledge the lived reality of YPLHIV in their community, and to address and work against harmful practices.

Finally, collaboration between biomedical, traditional, and religious health practitioners is pivotal to reduce risks and to improve the support of YPLHIV in a way that strengthens treatment adherence. The new empirical evidence presented in this study is an opening for new, contextualised communication and collaboration between religious, traditional, and medical health providers. It is an opportunity to reach beyond the level of simply talking about each other, and to start talking with each other. Some of the associations in this study were indirect and require some root cause analysis to determine the nature of influence. This can only be achieved in collaboration between religious, traditional, and medical stakeholders. The importance of talking together was also recognised at a high-level meeting with representatives of UNAIDS and leaders of different faith communities held on the sidelines of the WHO general assembly in September 2022 [23]. This meeting emphasized that the trust that followers place in religious and traditional leaders enables them to influence how people understand and react to HIV. Our study confirms this critical influence. The identification of significant links between certain religious affiliations and conceptions of HIV, treatment behaviour, and risk behaviours demonstrates the influence of traditional and religious leaders and health-practitioners on our study participants. We therefore welcome the decision of UNAIDS to strengthen its collaboration with faith communities. However, considering the significant links between certain religious affiliations and conceptions of HIV, and/or risk behaviour with treatment failure, the prevalence of opportunistic infections, and mental health risks in our study, we need to point out that faith communities’ influence may be a double-edged sword. While it may support and provide guidance in ways that increase wellbeing, treatment adherence, and health outcomes, the opposite is also true.

Thus, constructive collaboration between traditional, religious, and medical health providers may prove to be one of the decisive factors to better align what for now are too frequently disparate recommendations, expectations, and prescribed practices. This may prove to be a vital strategy toward achieving the eradication of AIDS by 2030.

74% of the YPLHIV in our study had an undetectable viral load of less than 50 copies per milliliter blood, suggesting that they respond and adhere well to treatment. However, 16% had a viral load higher than 200 copies per milliliter blood, suggesting treatment failure, and about 9% had mental health issues. This indicates that, while largely effective, the existing comprehensive care program nevertheless fails to cater adequately to the needs of all YPLHIV. To close this gap, this study identified specific predictive factors that can be utilized to inform new, religiously sensitive, and culturally more relevant measures of care that can reach those who are left behind.

According to UNAIDS, over 70% of the 1.7 million children living with HIV are in just 10 African countries [23]. Zimbabwe is among these. If these 10 countries further strengthened their efforts to end HIV in children, almost three-quarters of the challenge could be overcome. We believe that the findings of our study might be relevant for all these 10 African countries. Addressing the issues highlighted in our study, especially via the collaboration between health professionals and religious and traditional health practitioners and leaders, may signify an unprecedented potential to eradicate AIDS by 2030.

### Limitations

The quantitative study was conducted with clients of an ART program. This implies that they have not been hindered by religious/traditional beliefs or practices to engage in medical care. Thus, this study does not address the issue of certain religious groups that do not allow their members to seek medical help. This limitation, however, further underscores the importance of our findings relating to the significant intervention potential of traditional and religious leaders. If done correctly, the meaningful inclusion of all relevant stakeholders may even impact the lives of people that are currently beyond our reach. Furthermore, due to Covid-19 restrictions, the data collection was primarily done by phone calls. Whether respondents had the privacy to answer all questions freely could not be sufficiently verified. Data collectors assumed that the numbers of sensitive topic variables (especially the experience of sexual violence, drug use, and sexual behaviour) were underreported. All the more, the confirmation of the statistically relevant association of religious and traditional beliefs and practices on health outcomes and risk behaviour should be taken seriously and investigated further. We therefore encourage more studies in other sub-Saharan contexts to evaluate our findings.

## Conclusion

Religious and traditional practices and beliefs and religious affiliation significantly related to physical and mental health outcomes in our study population. Thus, the findings of this research provide evidence to support UNAIDS’ recognition of the pivotal role of faith communities in the efforts to end AIDS by 2030. The knowledge that religious and traditional beliefs and practices predict viral load results provides a basis for the development of more culturally sensitive, and more participatory, patient centered approaches of prevention and care, that include religious, traditional, and biomedical health care providers. These results might not only be relevant in Zimbabwe but also in other cultural and religious contexts, and have the potential to inform and support renewed unprecedented efforts to reach the goal of eradicating AIDS by 2030.

## Data Availability

The datasets used and/or analyzed during the current study are available from the corresponding author on reasonable request.

## Abbreviations

AIDS: Acquired Immune Deficiency Syndrome
ART: Antiretroviral therapy
ARV: Antiretroviral drug
CI: 95% Confidence Interval
HIV: Human Immunodeficiency Virus
ODK: Open Data Kit
OR: Odds Ratio
YPLHIV: Young People living with HIV
